# Gender-based *in vivo* comparison of culprit plaque characteristics and plaque microstructures using optical coherence tomography in acute coronary syndrome

**DOI:** 10.34172/jcvtr.2021.46

**Published:** 2021-11-01

**Authors:** Krishna Prasad, Sreeniavs Reddy S, Jaspreet Kaur, Raghavendra Rao k, Suraj Kumar, Vikas Kadiyala, Jeet Ram Kashyap, Garima Panwar

**Affiliations:** ^1^Department of Cardiology, Post Graduate Institute of Medical Education and Research (PGIMER), Chandigarh, 160012, India; ^2^Department of Cardiology, Government Medical College and Hospital, Sector 32, Chandigarh, 160030, India

**Keywords:** Acute Coronary Syndrome, Gender, Optical Coherence Tomography, Thin-cap Fibroatheroma

## Abstract

***Introduction:*** Women perform worse after acute coronary syndrome (ACS) than men. The reason for these differences is unclear. The aim was to ascertain gender differences in the culprit plaque characteristics in ACS.

***Methods:*** Patients with ACS undergoing percutaneous coronary intervention for the culprit vessel underwent optical coherence tomography (OCT) imaging. Culprit plaque was identified as lipid rich,fibrous, and calcific plaque. Mechanisms underlying ACS are classified as plaque rupture, erosion,or calcified nodule. A lipid rich plaque along with thin-cap fibroatheroma (TCFA) was a vulnerable plaque. Plaque microstructures including cholesterol crystals, macrophages, and microvessels were noted.

***Results:*** A total of 52 patients were enrolled (men=29 and women=23). Baseline demographic features were similar in both the groups except men largely were current smokers (*P* <0.001). Plaque morphology,men vs. women: lipid rich 88.0% vs. 90.5%; fibrous 4% vs 0%; calcific 8.0% vs. 9.5% (*P* = 0.64). Of the ACS mechanisms in males versus females; plaque rupture (76.9 % vs. 50 %), plaque erosion (15.4 % vs.40 %) and calcified nodule (7.7 % vs. 10 %) was noted (*P* = 0.139). Fibrous cap thickness was (50.19 ±11.17 vs. 49.00 ± 10.71 mm, *P* = 0.71) and thin-cap fibroatheroma (96.2% vs. 95.0%, *P* = 1.0) in men and women respectively. Likewise no significant difference in presence of macrophages (42.3 % vs. 30%, *P* = 0.76), microvessels (73.1% vs. 60 %, *P* = 0.52) and cholesterol crystals (92.3% vs. 80%, *P* = 0.38).

***Conclusion:*** No significant gender-based in-vivo differences could be discerned in ACS patients’ culprit plaques morphology, characteristics, and underlying mechanisms.

## Introduction

Coronary artery disease (CAD) constitutes the major cause of death and premature disability in developed countries and developing nations like India. Cardiovascular deaths account for 30% of all deaths globally, of which low and middle-income countries account for the majority (80%).^1^ Although the incidence of the acute coronary syndrome (ACS) is more in men; females have higher mortality and reinfarction rates.^2^ Females are usually associated with worse outcomes after ACS.^3,4^ Previous reports have shown smaller and less compliant conduit arteries in women compared to men contributing to ACS and worse outcomes.^5^ On the contrary, an in-vivo imaging study did not reveal any difference in gender in coronary artery size.^6^ Young women tend to have worse outcomes when they have obstructive coronary artery disease, whereas the older women have additional comorbidities which negatively impact the prognosis following acute myocardial infarction and its therapy or revascularization.^7^ The variations in clinical risk factors, comorbid conditions, hormonal profile, and anatomical disease patterns couldn’t explain the increased severity of coronary artery disease in females.^8^ Few pathological studies have shown that females have distinct atherosclerotic pathophysiology.^9^ However, there are few in vivo studies that analyzed gender variation in plaque characteristics in ACS patients.^7,10^ Optical Coherence Tomography (OCT), an intracoronary imaging technique for the identification of atherosclerotic plaques with an axial resolution of 10-20 μm is a better modality than intravascular ultrasound.^11^ The prime advantage of OCT is its ability to detect plaque morphology and helps in identifying vulnerable plaques or thin-cap fibroatheroma (TCFA).^11^ Pathological studies suggest that disruption of TCFA is the most common cause underlying ACS.^12^ Therefore, identification of TCFA prior to plaque rupture gives insight into the plaque stabilizing strategies. The purpose of the study was to a) compare culprit plaque morphology and characteristics between males and females with ACS by OCT b) identify gender differences in the mechanisms underlying ACS.

## Materials and Methods

### Study Population

This was a single-center, prospective, observational study undertaken at a tertiary care center in North India from June 2015 to August 2016. A total of 52 patients who presented with acute coronary syndrome (ACS) were part of the study after screening 71 patients. Unstable angina (USA) was diagnosed as recent onset rest angina lasting for 20 min with increased severity or crescendo pattern exertional angina, along with either ST-segment depression of 0.5 mV or T- wave inversion of 0.3 mV in any two contiguous leads and no rise in troponin. Non-ST elevation myocardial infarction (NSTEMI) was defined as ST and T wave changes in ECG accompanied by raised troponin.^13^ ST-elevation myocardial infarction (STEMI) was defined by the presence of typical angina, and ST-segment elevation of 1 mm in two adjacent limb leads or 2 mm in at least two contiguous precordial leads or presence of a new-onset left bundle branch block.^14^ Culprit vessel was identified using electrocardiography, region wall motion abnormalities on echocardiography and coronary angiography.

Participants were excluded if they had cardiogenic shock, left ventricular failure (Killip class 3 or 4), serum creatinine ≥2.5 mg/dL, patients who can’t tolerate dual antiplatelet therapy (DAPT), very small, tortuous coronary arteries, or if the images acquired were uninterpretable and thrombus aspiration prior to intravascular imaging. A written and informed consent was taken from all patients prior to enrolment in the study. The clinical risk factors for coronary artery disease like diabetes (DM), hypertension, smoking, hyperlipidemia, along with body mass index (BMI) of all patients and baseline investigations, were recorded. The protocol was in accordance with the Helsinki Convention, and the institutional ethics committee has approved the study.

### Coronary angiography

All patients were administered standard loading doses of aspirin, clopidogrel, or prasugrel prior to coronary intervention and imaging. Intravenous heparin (70-100 U/kg) was administered and titrated to maintain a therapeutic activated clotting time. The angiographic characteristics assessed were the identification of the culprit vessel, location of the culprit plaque, and the number of diseased vessels. Quantitative angiography was performed by Medis Q angio® XA 7.3 (Medis Medical imaging System, Leiden, the Netherlands). The PCI procedure was performed after intravascular imaging.

### OCT Acquisition and Analysis

**Figure 1:**
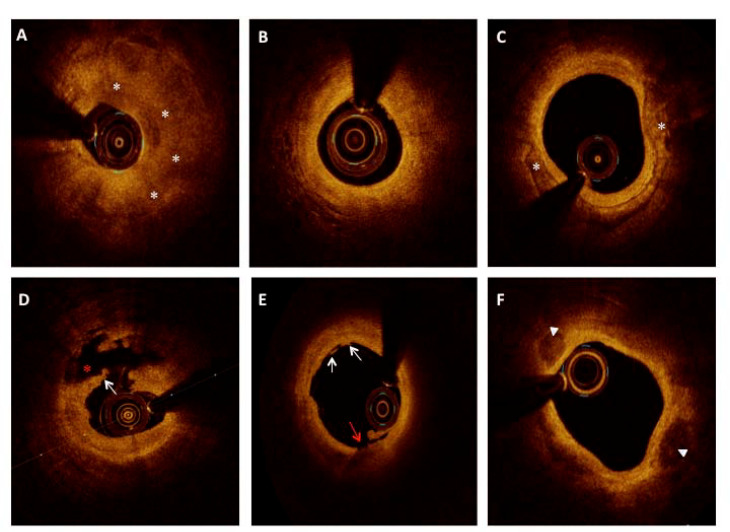
Representative OCT image of culprit plaque characteristics. Plaque morphology depicted in panel (A-C) and OCT mechanisms of ACS in panel (D-F). (A) Lipid-rich plaque (white asterisks indicate a large lipid pool). (B) Fibrous plaque. (C) Calcific plaque (white asterisks indicate calcium deposits). (D) Plaque rupture in a lipid-rich pool with disrupted fibrous cap (white arrow) and cavity formation (red asterisk). (E) Plaque erosion was identified as irregular luminal surface (white and red arrows) and intact underlying plaque (red arrow). (F) Calcified nodule detected with convex surface protruding into the lumen (white arrowheads)

**Figure 2:**
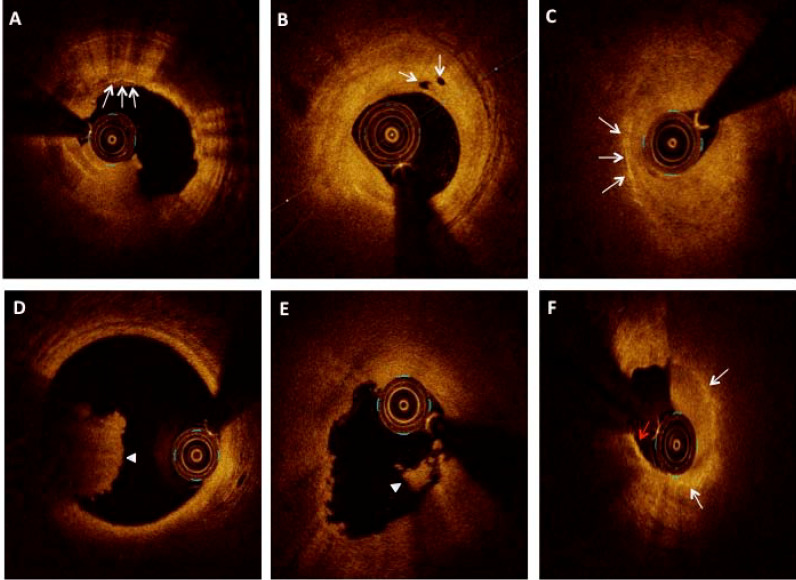
Representative OCT images of plaque microstructures and others. (A) Macrophages (white arrows). (B) Microvessels (white arrows). (C) Cholesterol crystals in a lipid-rich pool (white arrows). (D) Red thrombus (white arrowhead). (E) White thrombus (white arrowhead) along with plaque erosion with liminal irregular surface. (F) Thin-cap fibroatheroma 50 μm (red arrow) seen in a lipid rich plaque along with cholesterol crystals (white arrows).

After identification of the culprit vessel, an OCT catheter (C7-XR^TM^) St. Jude Medical^TM^, St. Paul, MN, USA) was passed over a 0.014” wire before any balloon dilatation. The pullback at a speed of 20 mm/sec was done after injection of contrast to get a blood-free field. All the images were analyzed offline by standard software (St. Jude Medical) by two independent investigators unaware of the patient’s clinical characteristics. The morphology of plaque was analyzed using the previously defined criteria.^11^ lipid-rich plaque was a signal poor along with ill-defined borders in the plaque encompassing > 90° in any cross-section (Figure 1A).^15^ The fibrous cap thickness measurement was taken at the thinnest portion in a lipid rich plaque. If the lipid-rich plaque contained an overlying fibrous cap with a minimum thickness of < 65μm, it was labeled TCFA (Figure 2F).^16^ Fibrous plaque was defined as a homogeneous component with low attenuation in a highly backscattered region. (Figure 1B). The calcific plaque was a low signal region with sharply defined margins (Figure 1C). The signal-rich, distinct, or confluent punctate regions with intensity greater than background speckle-noise were the macrophages (Figure 2A).^11^ Microvessels were the sharply delineated small signal-poor voids or tubular structures of 50–100μm diameter and their presence in at least three contiguous frames (Figure 2B).^17^ Thin linear regions of high-intensity signals situated in a fibrous or lipid-rich plaque were considered the cholesterol crystals (Figure 2C).^11^ Thrombus was defined as a mass protruding from the luminal surface towards lumen with > 250μm in diameter. Red thrombus was described as a highly backscattering and attenuated structure (Figure 2D), and white thrombus was a homogeneous, less backscattering, and low attenuation structure (Figure 2E). Culprit lesion was listed as a) plaque rupture, b) erosion, or c) calcific nodule. The calcified nodule was defined as a convex-shaped structure located superficially with high backscattering signals not exposed to the lumen and limited by a thin fibrous cap (Figure 1F). Plaque rupture was considered with identification of a cavity within the plaque along with fibrous cap discontinuity (Figure 2D). Plaque erosion was either termed, definite: when superficial thrombus and intact plaque, or probable: when the absence of thrombus but with irregular luminal surface or large thrombus precluding identification of underlying plaque and its evaluation but without any calcium or lipid in its vicinity (Figure 1E).^18,19^ The mean of the lipid arc was calculated by measuring the angle subtended by the lipid content in cross-sectional images using a protractor. Lipid index was estimated by multiplying the lipid arc average with the length calculated longitudinally of the lipid-rich component.^20^

### Statistical analysis

The baseline characteristics were displayed as mean ± standard deviation for continuous data and as frequencies for categorical data. The comparison of categorical variables was performed using the chi-square test or Fisher exact probability test, and continuous variables by the independent sample t-test. All the tests were two-sided, and a P-value<0.05 was considered statistically significant. The Statistical Package for the Social Sciences version (SPSS) version 26 IBM^TM^ corporation, USA was used for all analysis.

## Results

### Baseline patient characteristics

Out of 52 patients, 23 (44.2%) were females. The patients clinical characteristics are listed in Table 1. The study population had a mean age of 54.10 ± 9.50 years and was comparable in both genders (males: 53.83 ± 9.08 years, females: 54.43 ± 10.19 years). Twenty-nine (55.8%) patients had NSTEMI, and 23 (44.8%) had STEMI. Among males, 14 (48.3%) and in females, 9 (39.1%) had STEMI. Men were more often current smokers as compared to women (65.5 % vs. 4.3 %, *P* < 0.0001).

**Table 1:** Baseline Clinical Characteristics

**Variables**	**Males (n=29)**	**Females (n=23)**	***P*** ** value**
Age, years	53.8 ± 9.0	54.43 ± 10.1	0.882
Hypertension/ n, (%)	10/29 (34.5%)	9/23 (39.1%)	0.730
Diabetes mellitus/ n, (%)	5/29 (17.2%)	9/23 (39.1%)	0.077
Smoking/ n, (%)	19/29 (65.5%)	1/23 (4.3%)	<0.001
Family history/ n, (%)	13/29 (44.8%)	2/23 (8.7%)	0.004
Dyslipidemia/ n, (%) Total Cholesterol, mg/dL LDL, mg/dL HDL, mg/dL TG, mg/dL	23/29 (79.3%) 166.6 ± 41.6 100.4 ± 36.6 39.5 ± 5.3 133.1 ± 40.4	20/23 (86.9%) 166.2 ± 42.4 96.8 ± 34.4 41.0 ± 6.5 129.1 ± 35.8	0.546 0.977 0.714 0.358 0.708
Ejection Fraction (%)	49.6 ± 8.1	50.22 ± 5.32	0.77
STEMI/ n, (%)	14/29 (48.3%)	9/23 (39.1%)	0.510
NSTEMI/ n, (%)	15/29 (51.7%)	14/23 (60.9%)	0.510
Thrombolyzed/ n, (%)	8/29 (27.5%)	8/23 (34.7%)	0.577
Medications at admission Aspirin Statin	31.4 % 3.2 %	29.6 % 3.9 %	0.96 1.00

Abbreviations: NSTEMI, Non-ST elevation myocardial infarction; STEMI-ST elevation Myocardial Infarction; LDL, low-density lipoproteins; HDL, high-density lipoproteins; TG, triglycerides Data are presented as mean ± SD, or *n* (%)

### Angiographic Characteristics

Single vessel disease was predominantly seen in most of the patients and is no different in males and females (89.6 % vs. 86.9 %). The culprit vessel as left anterior descending artery (LAD) was considerably noted in both gender (68.9 % and 69.5 %). The majority of the plaques were distributed proximally (50%), with no significant gender difference in the plaque distribution. Quantitative angiographic variables like diameter at the tight stenosis, area of the stenosis, and reference vessel diameter were no different in males and females (Table 2).

**Table 2:** Angiographic Characteristics

	**Males (29)**	**Females (23)**	***P*** ** value**
**Culprit artery (%)**			
LAD	68.9%	69.5%	0.52
LCx	17.2%	4.3%	
RCA	13.7%	26.0%	
**Number of vessels involved (%)**			
SVD	89.6%	86.95%	0.24
DVD	6.89%	13.04%	
TVD	3.44%	0%	
**QCA Findings**			
Obstruction Diameter, mm	1.02 (0.88 – 1.15)	1.01 (0.84 – 1.17)	0.87
Reference vessel Diameter, mm	2.66 (2.38 – 2.93)	2.74 (2.41- 3.06)	0.74
Diameter Stenosis (%)	60.72 (56.19- 65.24)	62.61 (56.53- 68.68)	0.62
Area Stenosis (%)	83.50 (79.77 – 87.23)	84.67 (79.94- 89.39)	0.70
Length of the lesion, mm	11.53 (9.79 - 13.26)	12.25 (10.34 -14.15)	0.59

Abbreviations: QCA, quantitative coronary angiography; SVD, single vessel disease; DVD, double vessel disease; TVD, triple vessel disease; LAD, left anterior descending artery; LCx, left circumflex artery; RCA, right coronary artery Data are presented as mean ± SD, or *n* (%)

### OCT characteristics

**Figure 3:**
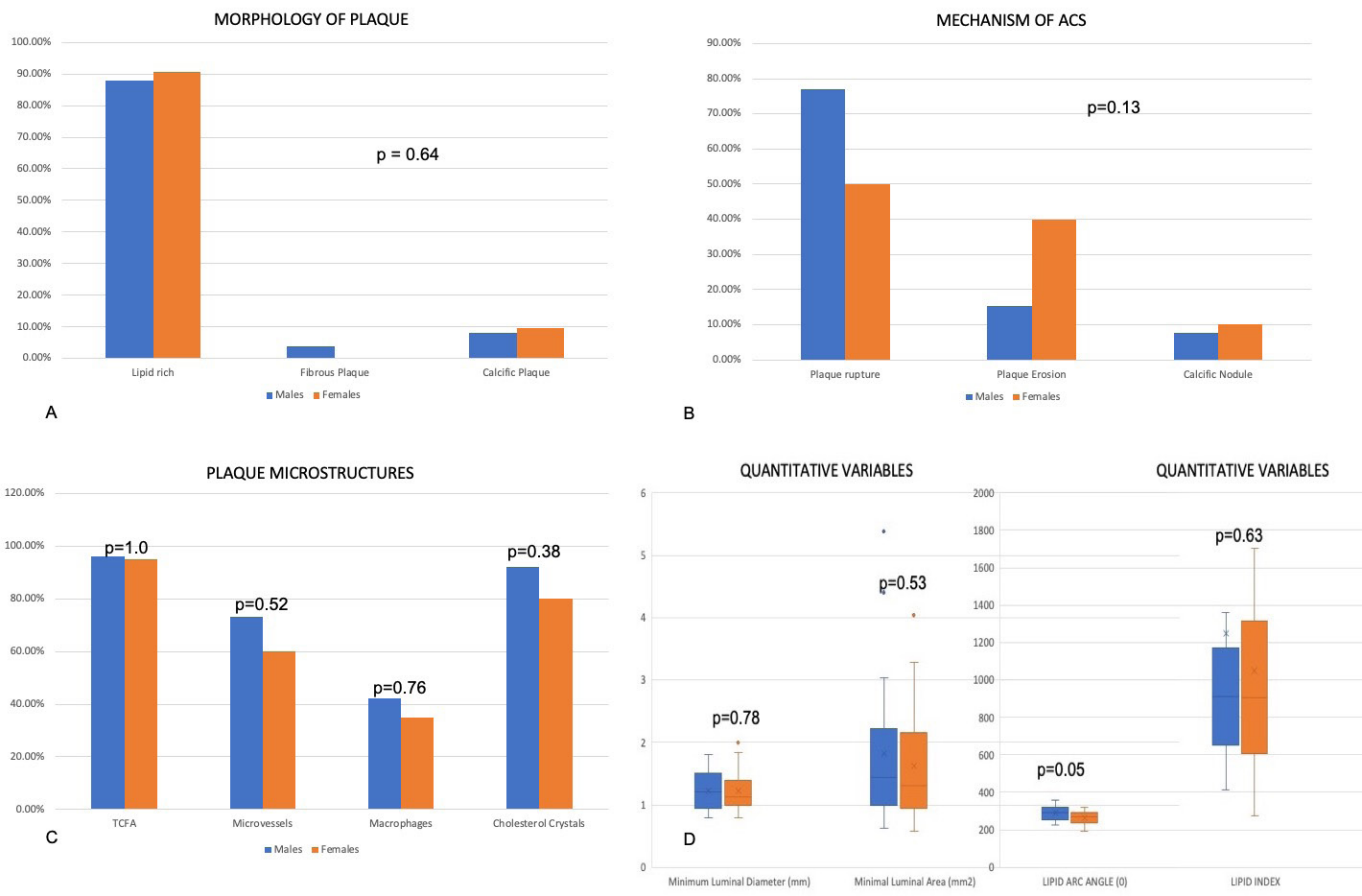
(A) Bar diagram showing frequency of various morphologies of plaque in males and females. (B) Bar diagram showing frequencies of various mechanisms of ACS in males and females. (C) Bar diagram showing frequencies of various microstructures in males and females. (D) Box Whisker plot showing distribution of various quantitative OCT variables in males and females

OCT characteristics and findings are shown in Table 3 and Figure 3. There was no gender distinction in the mechanism of ACS between males and females. Plaque rupture is the most common mechanism seen in 65.2% of all the patients, with no appreciable difference in males and females (76.9% vs. 50.0%). Although plaque erosion is higher in females numerically, the difference is not statistically significant (40 % vs. 15.4%, *P* = 0.13). Lipid-rich plaque is the most common morphology observed in 89.1 % of the patients. There was no difference in the morphology of the plaque among the gender groups [lipid rich 88.0% vs. 90.5%; fibrous 4% vs 0%; calcific 8.0% vs. 9.5% (*P* = 0.645)]. Males had a higher mean lipid arc than the females and had a trend for significance (289.61° ± 38.39° vs. 264.65° ± 37.44°, *P* = 0.05). The lipid index was not different between males and females (1250.44 ± 1554.42 vs. 1049.75 ± 654.77, *P* = 0.63). Likewise, no difference in TCFA was seen (96.2 % vs. 95.0 %, *P* = 1.0). The plaque microstructures including macrophages (42.3 % vs. 35.0%, *P* = 0.76), microvessels (73.1% vs. 60.0%, *P* = 0.52) and cholesterol crystals (92.3% vs. 80.0%, *P* = 0.38) did not reveal any statistical significance gender wise. Likewise, no significant difference in the distance of plaque rupture or plaque erosion site from ostium among the gender (18.40 ± 13.71 vs. 20.56 ± 9.570 mm, *P* = 0.52). The minimum luminal diameter (1.22 ± 0.30 mm vs. 1.21 ± 0.31 mm, *P* = 0.78); maximum diameter (1.72 ± 0.64 mm vs.1.54 ± 0.44 mm, p = 0.26); and minimal luminal area (1.80 ± 1.10 mm^2^ vs. 1.62 ± 0.89 mm^2^, *P* = 0.53) were no different among the males and females.

**Table 3:** OCT Characteristics

	**Males (n=29)**	**Females (n=23)**	***P*** ** value**
**Morphology of Plaque (%)**			
Lipid Rich	88.0%	90.5%	0.64
Fibrous	4%	0%	
Calcific	8%	9.5%	
**Mechanism underlying ACS (%)**			
Plaque Rupture	76.9 %	50.0%	0.139
Plaque Erosion	15.4 %	40 %	
Calcific Nodule	7.7 %	10 %	
**Thrombus**			
Red Thrombus	11.5%	5.0%	0.50
White thrombus	44.8%	25.0%	
**Plaque Microstructures (%)**			
Macrophages	42.3 %	35.0 %	0.76
Microvessels	73.1 %	60.0 %	0.52
Cholesterol Crystals	92.3 %	80.0 %	0.38
TCFA	96.2 %	95.0 %	1.0
Fibrous Cap thickness, μm	50.19 ± 11.17	49.00 ± 10.71	0.71
**Quantitative Analysis**
Minimum diameter, mm	1.22 ± 0.30	1.21 ± 0.31mm	0.78
Maximum diameter, mm	1.72 ± 0.64	1.54 ± 0.44	0.26
Mean diameter, mm	1.43 ± 0.41	1.39 ± 0.37	0.74
Minimal luminal area, mm^2^	1.8 ± 1.14	1.62 ± 0.89	0.53
Distance of plaque rupture or erosion from ostium, mm	18.40 ± 13.71	20.56 ± 9.570	0.52
Lipid Arc (Mean°)	289.61 ± 38.39	264.65 ± 37.44	0.05
Lipid Index	1250.44 ± 1554.42	1049.75 ± 654.77	0.63

Abbreviations: Lipid Index, mean lipid arc x lipid length; TCFA, thin-cap fibroatheroma; ACS, acute coronary syndrome; OCT, optical coherence tomography Data are presented as mean ± SD, or *n* (%)

## Discussion

Our study, an OCT-based in-vivo study, demonstrated that there were no significant gender differences in culprit plaque morphology, mechanisms of ACS, and plaque microstructures in patients presenting with ACS. This is the first in-vivo study in the Indian sub-continent to explore significant gender differences using high-resolution intracoronary imaging (OCT).

ACS occurs a decade early in the Indian population as compared to the western population.^21,22^ The age of onset of ACS in women was not different from that of men in this study (54.0 vs. 53.0 years). This is in contrast to other studies wherein the mean age of ACS occurrence was higher in females as compared to males (57.2 vs. 60.8 years in DEMAT registry).^23-25^

Angiographic features, including distribution of culprit plaque, the extent of disease, were studied to discern any difference among males and females. The disease’s extent was no different among the gender, with the single-vessel disease being the most common presentation. The culprit lesion was mainly LAD in both gender with no distinction with respect to vessel involvement. This is contrary to a study by Gudnadottir et al. wherein females with acute coronary syndrome are more likely to have less extensive disease.^25^ No difference could be ascertained in the quantitative coronary angiographic variables like diameter stenosis, area stenosis, length of the lesion and is in line with findings observed by Kang et al.^26^

Because of its high resolution, OCT helps identify the plaque morphology and culprit plaque characteristics and its mechanisms. No difference could be made in the plaque morphology between males and females. Lipid-rich plaque was seen in 88% of men and 90.5% of women. Other studies by Chia et al in 42 patients of acute coronary syndrome wherein lipid-rich plaques were observed in male versus females (79% vs. 89%; *P* = 0.84) and Rong et al in 212 patients in first STEMI (73.5% vs. 83.7%; *P*= 0.18) with no significant gender difference in culprit plaque morphology.^7,10^

The key underlying mechanisms leading to the acute coronary syndrome are plaque rupture, plaque erosion, or a calcified nodule. Many studies, both in-vivo and autopsy series, have demonstrated plaque rupture as the predominant cause of thrombosis leading to ACS.^27-29^ As shown previously, ruptured plaques are commonly observed in men with ACS, and plaque erosions are likely to be seen more frequently in women. This indicates that different mechanisms play a role in ACS development in men and women.^30,31^ In our study also plaque rupture was the predominant cause of ACS (65.2%). There was no difference in mechanisms underlying ACS between males and females, and these findings are in consonance with previous studies.^7,10,32^

Thus, based on our study, culprit plaque characteristics or mechanisms underlying ACS are less likely to explain the differences in outcomes. There are studies documenting gender differences in non-culprit plaque morphology including larger lipid pools, increased plaque erosion in females compared to males.^33-36^ However our study in culprit plaques did not show any variation in plaque morphology, as many times ACS occurs due to plaque rupture in a vulnerable plaque and is similar in both the genders. Gender variations in thrombotic milieu, hormonal influences and inflammatory response in ACS, disparities in age, risk factor profiles might contribute to variation in clinical presentation.^37,38^

Vulnerable plaques are prone to rupture.^39^ Presence of OCT-TCFA with fibrous cap thickness < 65μm is considered a prominent feature of a vulnerable plaque.^27,40,41^ Plaque microstructures that were analyzed included microvessels, macrophages, and cholesterol crystals. The presence of macrophages may predispose the plaque to rupture by the release of proteolytic components.^42^ Presence of cholesterol crystals have an incremental value in identifying vulnerable plaque by OCT.^43^ Previous pathological studies have demonstrated that cholesterol crystals may protrude through the fibrous cap and cause plaque rupture with thrombus formation.^44^ Microchannels or microvessels are associated with vulnerable plaques. Patients with microvessels have less fibrous cap thickness, more positive remodeling, and other features of vulnerability.^17^ There were no differences in plaque microstructures between males and females in our study. These findings are corroborated by similar studies where no difference could be recognized.^7,10^ The increased detection of cholesterol crystals (92.3% vs. 80.0%) and microvessels (73.1% vs. 60.0%) in the present study in males versus females was higher than in the previous report by Rong et al. wherein cholesterol crystal (26.0 vs. 30.6%) and microvessels (47.5 % vs. 51.0%) with no statistical difference gender-wise is a noteworthy finding. These plaque features are associated with plaque vulnerability and might contribute to a relatively early age onset ACS is an area of further research.

The study was observational with small sample size and is a major limitation. Nevertheless, a similar study with a lesser sample size could also not ascertain any gender differences.^7^ Some reports have questioned the OCT assessment and limitation of specific plaque characteristics like superficial calcification and macrophages. Patients with significant thrombus or slow flow were excluded as no thrombus aspiration was permitted as per protocol, which could alter the mechanism of ACS.

## Conclusion

The present study didn’t find any significant gender difference in culprit plaque characteristics and the mechanism underlying ACS in patients presenting with ACS. This study suggests that coronary plaque morphology and features in ACS per se are unlikely to explain the differences in outcomes observed in women. Further larger-scale studies are warranted to elucidate the underlying mechanisms.

## Acknowledgments

We thank the secretarial assistance of Ms Deepika and Mr Chander Bhushan.

## Competing interests

All authors have none to declare.

## Ethics approval

The study was approved by the Institutional Ethics Committee of Post Graduate Institute of Medical Education and Research, Chandigarh bearing reference number INT/IEC/2015/135.

## Funding

None.
